# Improvement of accumulated dose distribution in combined cervical cancer radiotherapy with deep learning–based dose prediction

**DOI:** 10.3389/fonc.2024.1407016

**Published:** 2024-07-08

**Authors:** Qi Fu, Xinyuan Chen, Yuxiang Liu, Jingbo Zhang, Yingjie Xu, Xi Yang, Manni Huang, Kuo Men, Jianrong Dai

**Affiliations:** ^1^ Department of Radiation Oncology, National Cancer Center/National Clinical Research Center for Cancer/Cancer Hospital, Chinese Academy of Medial Sciences and Peking Union Medical College, Beijing, China; ^2^ School of Physics and Technology, Wuhan University, Wuhan, China; ^3^ Department of Radiotherapy Technology, The Cancer and Tuberculosis Hospital, Jiamusi, China

**Keywords:** cervical cancer, combined radiotherapy, accumulated dose, deep learning, NTCP

## Abstract

**Purpose:**

Difficulties remain in dose optimization and evaluation of cervical cancer radiotherapy that combines external beam radiotherapy (EBRT) and brachytherapy (BT). This study estimates and improves the accumulated dose distribution of EBRT and BT with deep learning–based dose prediction.

**Materials and methods:**

A total of 30 patients treated with combined cervical cancer radiotherapy were enrolled in this study. The dose distributions of EBRT and BT plans were accumulated using commercial deformable image registration. A ResNet-101–based deep learning model was trained to predict pixel-wise dose distributions. To test the role of the predicted accumulated dose in clinic, each EBRT plan was designed using conventional method and then redesigned referencing the predicted accumulated dose distribution. Bladder and rectum dosimetric parameters and normal tissue complication probability (NTCP) values were calculated and compared between the conventional and redesigned accumulated doses.

**Results:**

The redesigned accumulated doses showed a decrease in mean values of V_50_, V_60_, and D_2cc_ for the bladder (−3.02%, −1.71%, and −1.19 Gy, respectively) and rectum (−4.82%, −1.97%, and −4.13 Gy, respectively). The mean NTCP values for the bladder and rectum were also decreased by 0.02‰ and 0.98%, respectively. All values had statistically significant differences (p < 0.01), except for the bladder D_2cc_ (p = 0.112).

**Conclusion:**

This study realized accumulated dose prediction for combined cervical cancer radiotherapy without knowing the BT dose. The predicted dose served as a reference for EBRT treatment planning, leading to a superior accumulated dose distribution and lower NTCP values.

## 1 Introduction

The combination of external beam radiotherapy (EBRT) and brachytherapy (BT) is a standard treatment for cervical cancer. The EBRT part of the treatment aims to treat the whole pelvis, including the tumor and the lymph nodes at risk. The BT part of the treatment aims to boost the residual tumor in multiple fractions. Ideally, all treatment parts should be combined for dose optimization and evaluation. However, EBRT and each fraction of BT are optimized independently in current clinical practice. This approach may result in a less than optimal accumulated dose distribution that limits the dosimetric advantage of combined radiotherapy. If treatment planners knew the possible accumulated dose distribution before EBRT treatment planning, then they could modify the dose to the organs at risk (OARs) from EBRT and leave more OAR dose space for BT. Additionally, the independent dose optimization results in multiple dose distributions associated with different planning images, which creates difficulties for total dose evaluation. Currently, radiation oncologists (ROs) only assess total dose to OARs by summing the dose-volume parameters of EBRT and BT. This approach is a “worst case assumption” and usually does not reflect the actual doses received by OARs, especially in the case of significant dose gradients from external beam boosts. If a concrete accumulated dose distribution could be predicted before treatment initiation, then ROs could estimate the curative effect and radiation toxicity, thereby helping make better treatment decisions. Therefore, accumulated dose prediction has potential benefits for both dose optimization and total dose estimation.

Deep learning is an advanced technique with the capability to build robust prediction models in complex feature extraction. Recently, deep learning has rapidly developed and been successfully implemented in medicine and radiotherapy applications, such as toxicity prediction, automatic segmentation, synthetic image generation, and quality assurance (1–6). A convolutional neural network (CNN) is a deep learning algorithm that automatically extracts multi-level features from input data, resulting in more concise and effective prediction. Research groups have developed prediction models based on CNNs and demonstrated their success in three-dimensional dose distribution prediction (7–12). Furthermore, knowledge-based planning (KBP) utilizing prior patient treatment plans to make dosimetric predictions for new patients has shown promise in assisting treatment planning. Studies have proved that KBP can improve plan quality consistency (13–18). For cervical cancer, these methods have been applied separately to EBRT or BT (19–32).

For accurate image registration and dose accumulation, a deformable image registration (DIR) method has been developed. This method accounts for anatomic variations and provides a spatial transformation relationship between volume elements of corresponding structures in different images. This transformation can be applied to dose distributions, thereby enabling dose accumulation with high-precision. Recent studies have proved the feasibility of DIR for evaluating accumulated doses in combined radiotherapy. However, DIR remains a challenge for combined cervical cancer radiotherapy as large and complex deformations are likely to occur in the pelvic cavity (33–38).

In this study, we attempted to predict accumulated dose distribution to assist treatment planning and obtain a superior dose distribution. We accumulated the dose distributions from EBRT and BT using commercial DIR and used them to train a CNN-based dose prediction model. Then, we quantitatively evaluated the prediction performance. The practicability of the predicted accumulated dose in clinic was verified by redesigning EBRT plans.

## 2 Materials and methods

### 2.1 Patient data

A total of 30 cervical cancer patients who underwent 25 fractions of EBRT using volumetric-modulated arc therapy (VMAT) followed by more than 3 fractions of high-dose-rate BT were selected in this study. CT scans were performed with a Brilliance CT Big Bore (Philips, Amsterdam, Netherlands) or a Somatom Definition AS 40 (Siemens Healthcare, Forchheim, Germany) with 512 × 512 matrix. The CT slice thickness was 5 mm for EBRT and 3 mm for BT, respectively. The clinical target volume (CTV) of EBRT was the gross tumor volume (GTV), the entire uterus and cervix, the lymph nodal region at risk (GTVnd), and part of the bladder and rectum. A margin of 5 mm was applied around the CTV and GTVnd to create the planning target volume (PTV) and planning GTVnd (PGTVnd). The PTV was prescribed with 45 Gy or 50 Gy, and the PGTVnd was boosted by 10 Gy to 15 Gy. Normal tissues, including the rectum, bladder, intestine, colon, sigmoid, pelvic bone, and right and left femur head, were also delineated for planning and dose evaluation. The EBRT VMAT plans were designed using Pinnacle v9.1–16.2 (Philips Radiation Oncology Systems, Fitchburg, WI, USA) and delivered using double full arcs with 6-MV X-rays. All plans were optimized to ensure that at least 95% of the target volumes received the prescription dose (PD) while minimizing doses to OARs. To manage the deformation caused by different organ filling, each patient was requested to empty their bladder and rectum, and drink 800 mL of water 40 min before undergoing treatment. Cone-beam CT was used to verify their anatomical situation.

The target volume and OARs for BT were delineated on the planning CT according to Groupe Européen de Curiethérapie (GEC)- European SocieTy for Radiotherapy & Oncology (ESTRO) recommendations, including the high-risk CTV (HR-CTV), rectum, bladder, sigmoid, and bowel. MRI at diagnosis acquired before the first BT fraction was served as a reference. All BT plans were designed using Oncentra Brachy v4.6 (Elekta Brachytherapy, Veneedal, The Netherlands) and delivered using tandem/ovoid (T/O) applicators or T/O applicators with interstitial needles. A Flexitron afterloader unit with an ^192^Ir source was used for the BT treatment. The activation step was 2 mm. The source dwell times for BT plans were optimized by inverse planning simulated annealing and then manually adjusted on the basis of OAR dose constraints. The fractional PD was prescribed to cover 90% of the HR-CTV and normalized to 6 Gy for a high-precision dose prediction.

### 2.2 Method description

**Figure 1:**
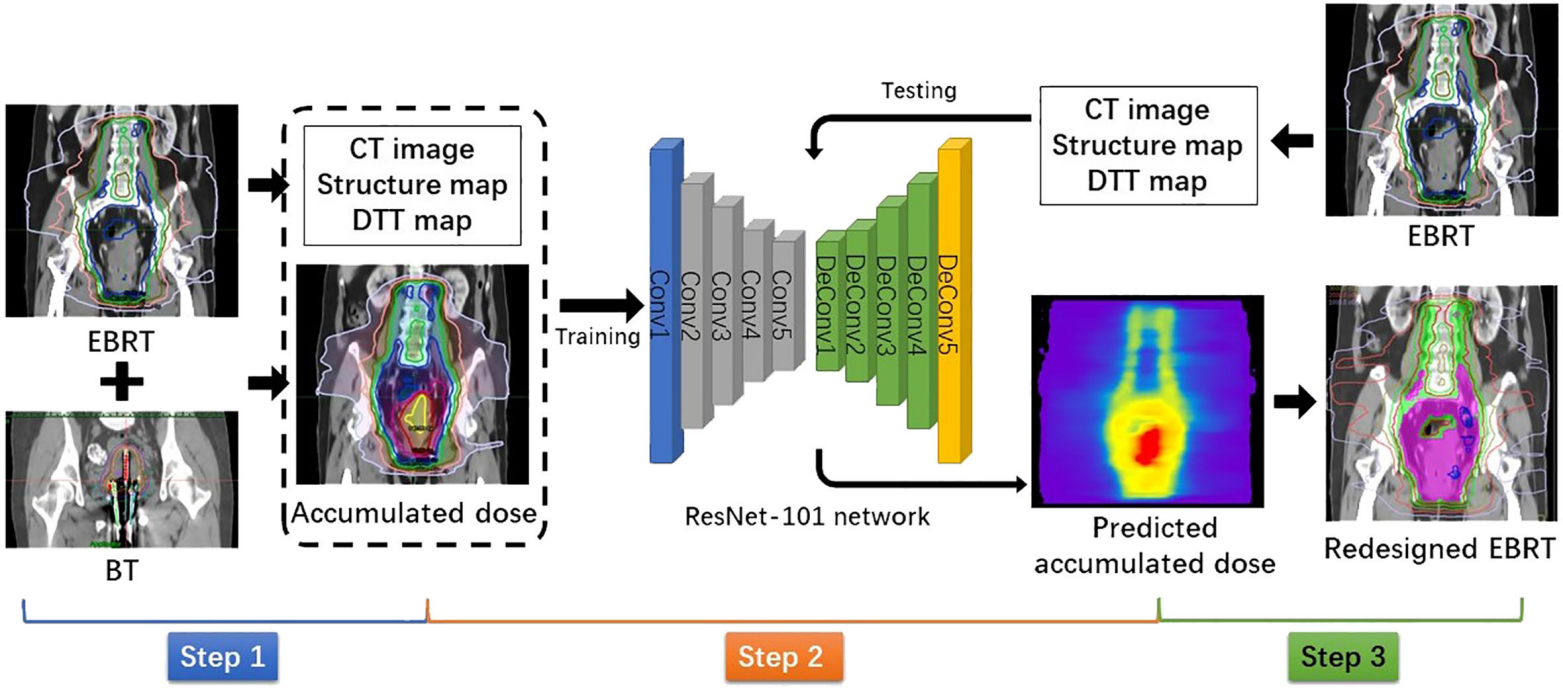
Flowchart showing dose accumulation, dose prediction, and plan redesign steps.

The flowchart shown in **Figure 1** illustrates the method of this study. It can be divided into three steps: dose accumulation, dose prediction, and plan redesign.

**Step 1: Dose accumulation**

To accumulate the dose distributions from EBRT and BT, all doses were converted into equivalent doses in 2 Gy fraction (EQD2) using the linear quadratic model with α/β = 10 Gy for tumors and α/β = 3 Gy for normal tissues. Due to limited conditions of our hospital, CT-guided BT was only performed for the first fraction. Thus, we could only magnify the first fractional BT dose four times to simulate the total BT dose. Commercially available DIR software MIM Maestro (MIM Software, Cleveland, OH, USA) was used to deform and sum the BT dose to the referenced EBRT dose to obtain the accumulated dose distribution. For a high DIR accuracy, we adopted a hybrid DIR method combining contour-based and intensity-based DIR. The contours used for DIR were the bladder, rectum, and the whole uterus and vagina (U + V). The purpose of delineating the U + V is to include the BT applicator and packing material. The CT numbers of these contours were, respectively, overridden to a certain value to ensure consistent intensities in both images. For the purpose of dose prediction, all accumulated doses were interpolated into the same pixel size with corresponding EBRT CT images.

**Step 2: Dose prediction**

The pixel-wise dose distribution prediction used a ResNet-101–based deep learning model, which has been introduced in our previous study (39). In this study, we also used combined anatomic maps as three-channel inputs, including the planning CT images of EBRT, corresponding structure maps, and distance maps. In the structure maps, the voxels in each target and OAR were assigned with a unique label. For voxels in the overlap, their label values were summed to ensure uniqueness. The voxels in the rest of the body and out of the body were labeled as 1 and 0, respectively. To improve dose prediction accuracy, we introduced a distance map as an input for the model. It was defined as the minimum distance from the PTV surface to each voxel of normal tissue outside the PTV, called the distance-to-target volume (DTT). In each slice of the DTT map, each voxel corresponded to a label value of DTT, and voxels inside the PTV and outside the body were labeled as 0. The outputs for the deep learning model were the corresponding accumulated dose maps. Due to the limited number of available datasets, a fivefold cross-validation was used to test prediction model performance. The prediction performance was quantitatively evaluated by (1) the voxel-wise mean absolute error (MAE) and (2) the dice similarity coefficient (DSC) of isodose volumes. Isodoses from 10 Gy to 160 Gy were evaluated with a 10-Gy interval.

**Step 3: Plan redesign**

**Figure 2:**
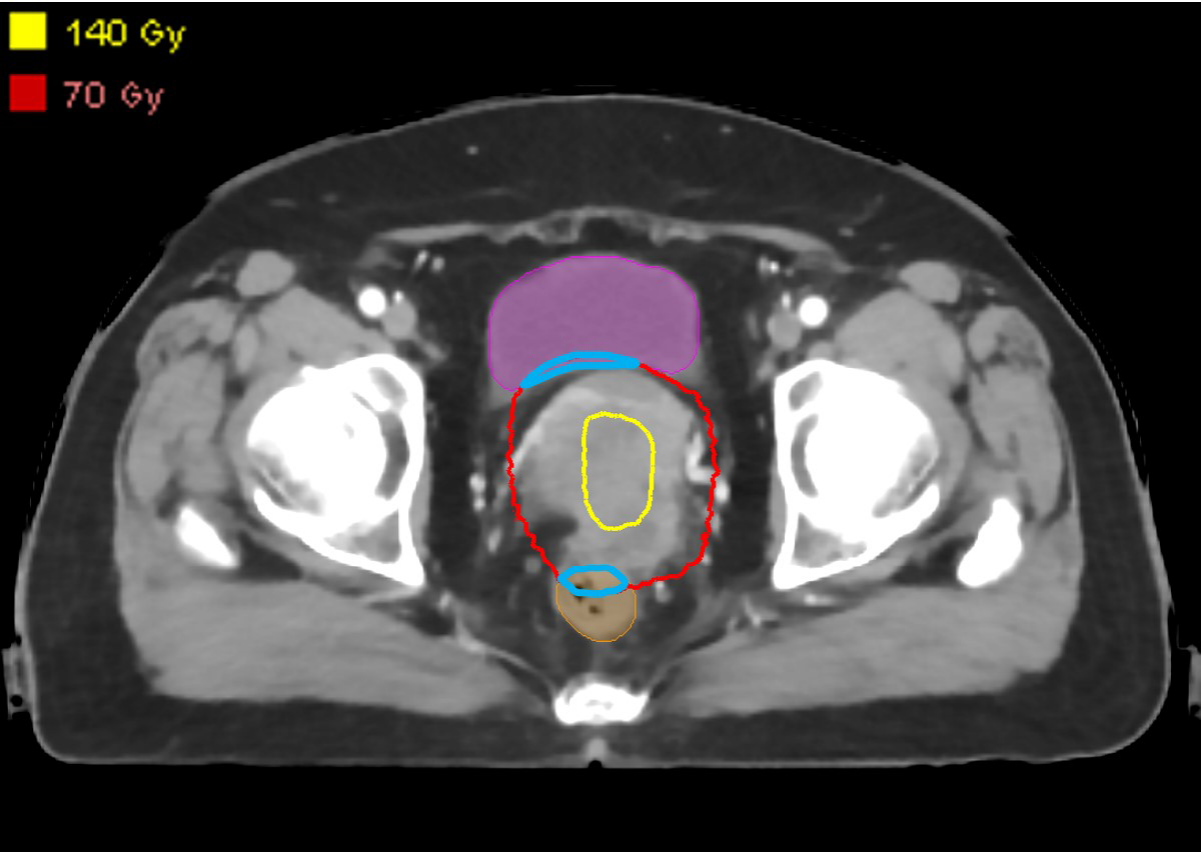
Overlaps (blue contours) between the predicted isodose volume of 70 Gy and the bladder/rectum.

The predicted accumulated dose distribution was used as a reference to redesign EBRT VMAT plans. As the BT dose is distributed within the cervix, the accumulated dose region is contained in the EBRT target. In terms of the region outside the EBRT target, the result of accumulated dose prediction would be similar to that of pure EBRT dose prediction. Therefore, the dosimetric parameters obtained from the predicted accumulated dose distribution could be used as optimization objectives for most normal tissues. However, as the bladder and rectum are partly included in the EBRT target, they are usually close to the high-dose region. As shown in **Figure 2**, the overlaps between the predicted isodose volume of 70 Gy and the bladder/rectum were created as a contour. For the redesigned plans, we minimized the dosimetric hotspots from occurring in this contour.

### 2.3 Clinical validation

To test the role of the predicted accumulated dose in clinic, one physicist designed an EBRT VMAT plan using the conventional method without knowing the predicted dose and then redesigned a new EBRT VMAT plan using the proposed method. The two resulting EBRT dose distributions were separately accumulated with the BT dose for comparison. Critical dosimetric parameters of the bladder and rectum (V_50_, V_60_, and D_2cc_) were recorded. Normal tissue complication probability (NTCP) values were calculated using the Lyman–Kutcher–Burman model. The estimated parameter values for the rectum and bladder were D50 = 80 Gy, m = 0.15, and n = 0.12 and D50 = 80 Gy, m = 0.11, and n = 0.50, respectively (40). The statistical significance of the results was proven with a Wilcoxon signed-rank test at 5% level significance.

## 3 Results

### 3.1 Dose prediction performance

**Figure 3:**
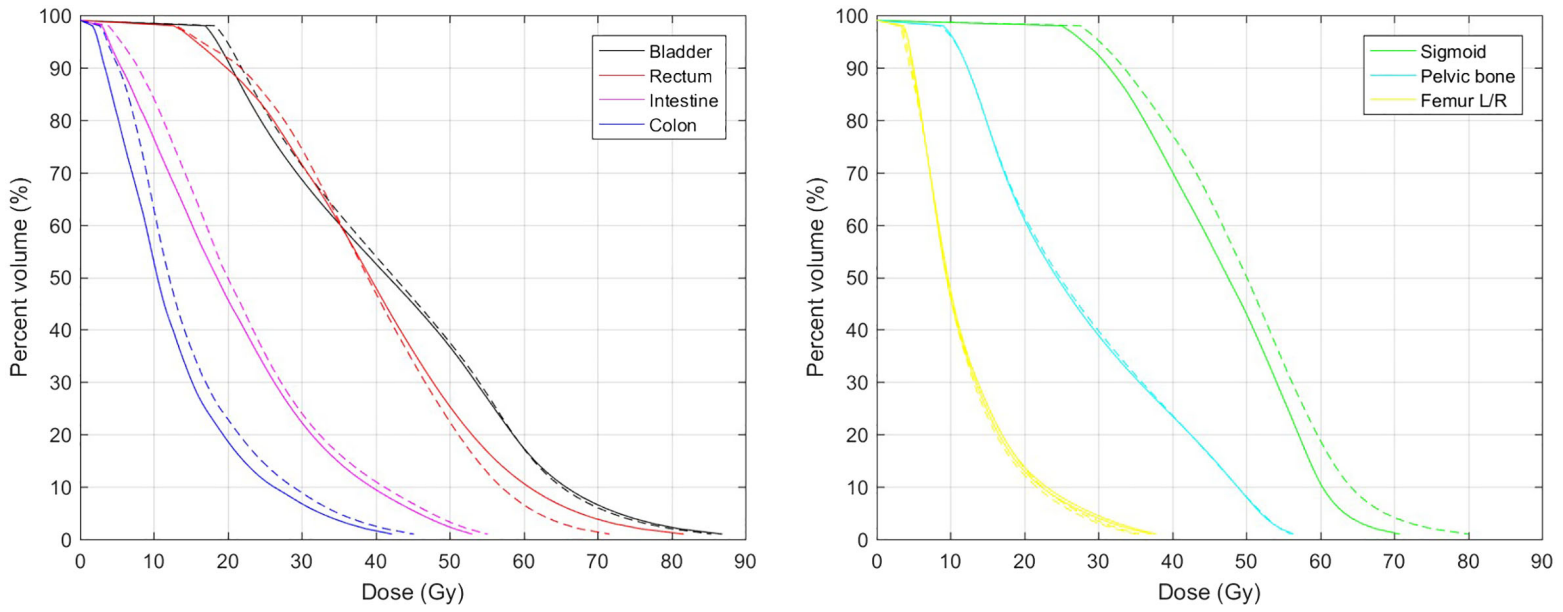
Mean DVHs of OARs. Solid and dashed lines represent actual and predicted DVHs, respectively.

The mean MAE between predicted and actual dose distributions were as follows: 3.73 ± 1.58 Gy for the whole body, 3.27 ± 1.06 Gy for the whole body without PTV, 6.55 ± 3.10 Gy for the bladder, 8.05 ± 4.95 Gy for the rectum, 4.10 ± 1.88 Gy for the intestine, 3.67 ± 2.01 Gy for the colon, 5.06 ± 3.10 Gy for the sigmoid, 3.48 ± 1.28 Gy for the pelvic bone, 2.71 ± 1.52 Gy for the femur head, 4.96 ± 3.32 Gy for the cord, and 2.75 ± 1.56 Gy for the kidney. Because the bladder, rectum, and sigmoid were close to the high-dose region, their mean MAE was larger than that of other OARs. **Table 1** presents the results of the DSC of isodose volumes between predicted and actual dose distributions. For an isodose less than or equal to the PD of EBRT, the mean DSC was high, ranging from 0.87 to 0.92, whereas the mean DSC of isodose volumes higher than the PD of EBRT was low and decreased with increasing isodose, ranging from 0.73 to 0.49. For the isodose volume of the summed PD of EBRT and BT (76.25/82 Gy_EQD2_), the mean DSC was approximately 0.7. Compared to the actual dose distribution, the predicted dose distribution exhibits characteristics of a more homogeneous dose in the target, lower dose gradient, and indistinct boundaries between the target and normal tissues. Consequently, the predicted volumes for low isodoses were slightly larger, whereas those for high isodoses were smaller compared to the actual volumes. This observation is also evident in **Figure 3** that illustrates a comparison between the predicted and actual DVHs for all OARs. Furthermore, the predicted and actual spatial locations of the high-dose regions of the bladder and rectum were similar. For the bladder, the high-dose region often occurs in the trigonum vesicae. In the rectum, it is often located near the upper cervix segment.

**Table 1:** Mean DSC of isodose volumes between predicted and actual dose distributions.

Isodose (Gy)	DSC	Isodose (Gy)	DSC
10	0.90 ± 0.01	90	0.67 ± 0.09
20	0.87 ± 0.02	100	0.64 ± 0.09
30	0.89 ± 0.01	110	0.61 ± 0.08
40	0.92 ± 0.01	120	0.59 ± 0.08
50	0.90 ± 0.04	130	0.56 ± 0.09
60	0.73 ± 0.10	140	0.54 ± 0.09
70	0.73 ± 0.09	150	0.51 ± 0.10
80	0.70 ± 0.09	160	0.49 ± 0.10

### 3.2 Redesigned plan evaluation

**Figure 4:**
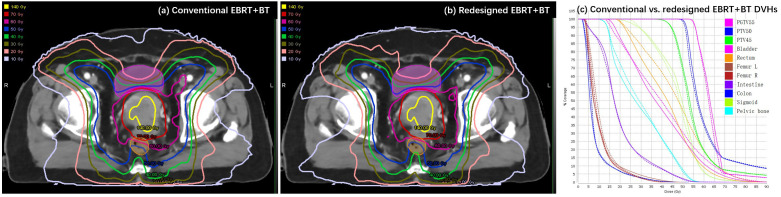
Example of **(A, B)** dose distributions and **(C)** DVHs for the conventional (dashed lines) and redesigned (solid lines) EBRT + BT.

**Table 2** shows the dosimetric parameters of the bladder and rectum for the conventional and redesigned EBRT + BT. Compared with the conventional EBRT + BT, the mean V_50_ and V_60_ of the redesigned EBRT + BT for the bladder decreased by 3.02% and 1.71%, respectively, whereas those for the rectum decreased by 4.82% and 1.97%, respectively. All values showed significant differences (p < 0.001). The mean D_2cc_ of the bladder and rectum for the redesigned EBRT + BT were also lower than those for the conventional EBRT + BT (−1.19 Gy and −4.13 Gy, respectively). The rectal D_2cc_ showed statistical significance (p < 0.001). **Figure 4** shows typical dose distributions and DVHs of the conventional and redesigned EBRT + BT for a single patient.

**Table 2:** Dosimetric parameters of bladder and rectum for the conventional and redesigned EBRT + BT.

	Bladder			Rectum		
	Conventional	Redesigned	P value	Conventional	Redesigned	P value
V_50_ (%)	34.47 ± 7.51	31.46 ± 7.28	<0.001	16.92 ± 10.40	12.10 ± 8.23	<0.001
V_60_ (%)	14.35 ± 5.82	12.64 ± 5.42	<0.001	6.25 ± 4.96	4.28 ± 3.87	<0.001
D_2cc_ (Gy)	85.56 ± 9.89	84.37 ± 9.18	0.112	61.82 ± 10.90	57.69 ± 10.46	<0.001

### 3.3 NTCP results

**Figure 5:**
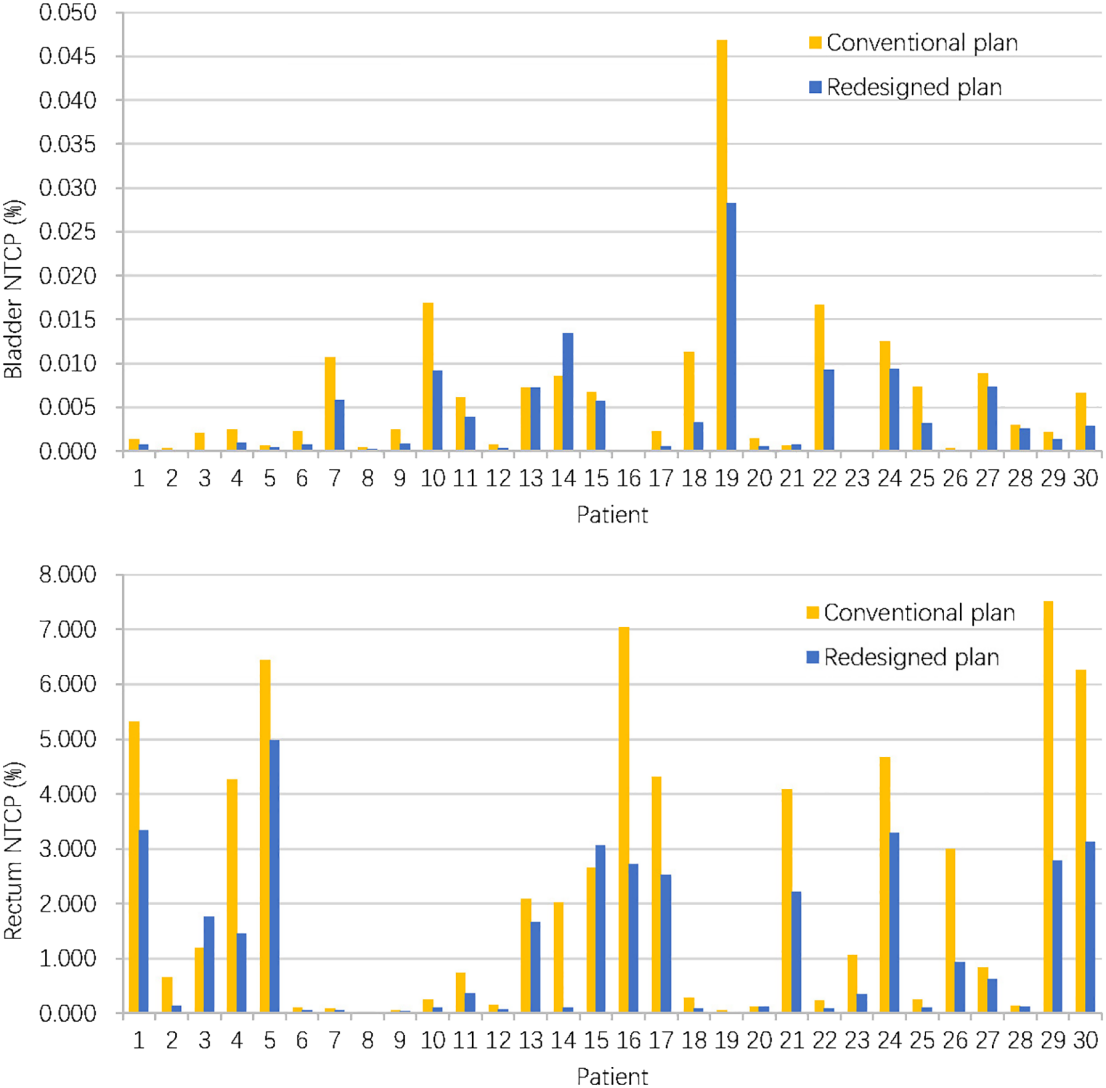
Bladder and rectum NTCP values for conventional and redesigned EBRT + BT.

When considering NTCP, the redesigned accumulated doses resulted in a decrease of NTCP for the bladder (0.04 ± 0.06‰ vs. 0.06 ± 0.09‰, P = 0.004) and rectum (1.21 ± 1.39% vs.2.19 ± 2.41%, P < 0.001). **Figure 5** compares bladder and rectum NTCP values for conventional and redesigned EBRT + BT for each patient.

## 4 Discussion

To address dose optimization and evaluation difficulties associated with separate implementation of EBRT and BT, we predicted the accumulated dose distribution using a combination of DIR and deep learning techniques. High-precision DIR is particularly challenging in the pelvic region due to large and complex deformations caused by tumor shrinkage, different organ filling, bowel gas, and the presence of a BT applicator and vaginal packing. Thus, we adopted an advanced hybrid DIR method, which could minimize both the intensity differences between the two images and the differences between corresponding contour surfaces. Additionally, we delineated the U + V and used it as a registration contour. As an important anatomy in the whole pelvis, it could not only solve the issue of inconsistent intensity between the EBRT and BT images but also help to improve the DIR accuracy. After the DIR, the mean DSC for all contours reached 0.8–0.9, which satisfied the accuracy requirement recommended by AAPM TG-132 report (41).

Although dose prediction has been successfully realized in studies, the accumulated dose prediction faces new challenges. The input data for deep learning were all based on EBRT. However, the output data were accumulated in dose maps correlated with both EBRT and BT. This led to an uncertain location of the predicted accumulated dose region in the EBRT targets. Consequently, the DSC of the isodose volume for the summed PD was relatively low for our initial dose prediction. To solve this problem, we added the U + V to the structure maps. Although it is an extra delineated structure used for DIR, it could specify the location of the BT dose. With this structure, the DSC of the isodose volumes was increased by approximately 0.05 for the 100%, 150%, and 200% summed PD.

However, the accuracy of the accumulated dose prediction is still affected by many factors, including the DIR method, the number of training data, and the various types of applicators used in BT. It can be observed from **Table 1** that there was a significant decrease in mean DSC when the isodose exceeded the PD of EBRT. This suggests that, while the current model performs well in predicting the EBRT dose part, improvements are required for accurately predicting the BT dose part. Enhancing accuracy in BT dose prediction may be achieved through adopting a more powerful DIR method, increasing the training data, and using a consistent BT applicator. Another limitation of this study is that we used the dose of the first BT fraction to represent the total BT dose due to the limited clinical data. This led to a deviation from the actual BT dose indeed. However, the treatment planning only needs the general scope of high-dose region as a reference, which is confirmed to be basically consistent among BT fractions (42) and could be provided by the first BT fraction. Moreover, multiple DIRs need to be performed if accumulating multiple BT doses. Our approach could avoid multiple errors caused by multiple DIR. The intra-fraction variations in the tumor and OARs were also not taken into account in this study. In fact, these variations will result in a deviation between the predicted dose and the actual dose (43, 44). The impact of these variations to different degrees on the actual effectiveness of the dose prediction model needs to be specifically studied in the future.

Treatment planning is a complex process with a large amount of optimization parameters to adjust based on the skill and experience of the planner. In recent years, KBP is increasingly used to improve plan quality and consistency. In previous literatures, the dose predictions used for KBP mainly focused on EBRT or BT alone (19–32). Li et al. applied a commercial KBP system to control EBRT plan quality in a clinical trial. They reported that the mean NTCP for gastrointestinal toxicity was lower for KBP plans compared to validation-set plans (48.7% vs. 53.8%, P < 0.001). Chen et al. used CNN to predict a patient-specific set of IMRT objectives based on overlap volume histograms (OVHs). They showed that the V_40_ of the bladder and rectum decreased by 6.3% and 12.3% compared to that of manual plans. In addition to EBRT, a research group applied knowledge-based dose prediction to BT (19, 24, 25). Their latest results showed that the differences between actual and predicted D_2cc_ were −0.17± 0.67 Gy, −0.04 ± 0.46 Gy, and 0.00 ± 0.44 Gy for the bladder, rectum, and sigmoid, respectively. Reijtenbagh et al. also used machine learning models to predict D_2cc_ to OARs using OVHs for identifying plans that may require further optimization. The models achieved mean squared errors ranging from 0.13 Gy to 0.40 Gy (31). Furthermore, dose prediction models have also been used to assess intra-fractional dose variations in OARs (27). To our knowledge, the accumulated dose of EBRT and BT is currently used only for predicting toxicity prediction and not for KBP (45, 46). However, for this combined radiotherapy, dose prediction for EBRT alone is not sufficient to guide EBRT treatment planning and BT doses should be taken into account to minimize the overall risk of toxicity. However, the problem is that EBRT is usually implemented before BT and the BT dose is unknown. This study realized accumulated dose prediction without knowing the BT dose, thereby allowing the BT dose to be taken into account in EBRT treatment planning. With the predicted dose, planners could set optimization objectives more appropriately to improve the consistency of plan quality. More importantly, the prediction could indicate an overlap between the high-dose region and OARs. This could help planners further decrease the EBRT dose to OARs, leave more OAR dosimetric space for BT, lower the overall OAR dose, and achieve an optimal accumulated dose distribution. For the redesigned EBRT plans, we minimized the hotspots occurred in the overlaps between the predicted high-dose region and the bladder/rectum. The results verified the redesigned EBRT + BT had lower total doses to the bladder and rectum. However, this study did not consider the sigmoid and bowel as they are too complex to be accurately registered. Furthermore, we only used the predicted accumulated dose for the EBRT planning. Ideally, it can be helpful for both EBRT and BT planning. This may be our next research direction.

Accumulated dose prediction could also be helpful to ROs. In current clinical practice, the actual accumulated dose for combined radiotherapy is not entirely clear or visible. Usually, tumor dosages are determined on the basis of the guidelines and combined with personal experience. Total OAR doses are evaluated on the worse case assumption. Accumulated dose prediction provides a final dose distribution before treatment initiation. It could help ROs, especially young and inexperienced ROs, in making treatment decisions, delineating targets, guiding the insertion of the BT applicator, and predicting the risk of toxicity. This effect could be further explored in future work.

## 5 Conclusion

In this study, we realized accumulated dose prediction for combined cervical cancer radiotherapy without knowing the BT dose. EBRT treatment planning referencing the predicted accumulated dose distribution could improve accumulated dose distribution and decrease OAR NTCP values. The predicted dose could also help ROs estimate the total doses to OARs and make better treatment decisions.

## Data availability statement

The raw data supporting the conclusions of this article will be made available by the authors, without undue reservation.

## Ethics statement

The studies involving humans were approved by the Ethics Committee of National Cancer Center/Cancer Hospital, Chinese Academy of Medial Sciences and Peking Union Medical College (protocol code 2023020809270002 and 8 February 2023). The studies were conducted in accordance with the local legislation and institutional requirements. Written informed consent for participation was not required from the participants or the participants’ legal guardians/next of kin in accordance with the national legislation and institutional requirements.

## Author contributions

QF: Writing – original draft, Writing – review & editing, Conceptualization, Investigation, Methodology, Project administration, Data curation, Formal analysis, Visualization. XC: Conceptualization, Data curation, Investigation, Methodology, Software, Writing – original draft, Formal analysis, Resources. YL: Writing – original draft, Formal analysis, Investigation, Resources, Software. JZ: Writing – original draft, Data curation, Formal analysis, Investigation. YX: Writing – original draft, Methodology, Supervision, Validation. XY: Data curation, Supervision, Validation, Writing – original draft. MH: Data curation, Supervision, Validation, Writing – original draft. KM: Conceptualization, Funding acquisition, Project administration, Supervision, Writing – original draft. JD: Project administration, Supervision, Writing – original draft.
